# Study on the Microstructure and Properties of AISI 304 Stainless Steel Corrugated Pipes by Aging and Solution Treatments

**DOI:** 10.3390/ma18061387

**Published:** 2025-03-20

**Authors:** Xiang Zhao, Anheng Wang, Jianbin Wang, Chuanwen Ling, Xiaolong Gui

**Affiliations:** School of Mechanical and Automotive Engineering, Anhui Polytechnic University, Wuhu 241000, China; zxahpu@126.com (X.Z.); wjb@ahpu.edu.cn (J.W.); cwlsh904@163.com (C.L.); guixiaolong2024@163.com (X.G.)

**Keywords:** corrugated tube, aging treatment, solution treatment, microstructure, mechanical property

## Abstract

This article focuses on the microstructural evolution and mechanical property changes of AISI 304 austenitic stainless steel corrugated pipes after aging treatment and solution treatment. The influence of different heat treatment processes on the microstructural evolution, second phase precipitation behavior, mechanical properties, and corrosion resistance of corrugated pipes was analyzed through metallographic microscopy (OM), scanning electron microscopy (SEM), electron backscatter diffraction (EBSD), fatigue testing, hardness testing, and corrosion resistance experiments. The results showed that after aging treatment at 600 °C, carbides precipitated at the grain boundaries and twin boundaries of the corrugated tube, leading to corrosion behavior. The average microhardness value was 266.08 HV, and the work hardening problem of the corrugated tube was not improved. After solution treatment at 1050 °C, a single-phase austenite structure was obtained in the corrugated tube, and the carbides at the grain boundaries were completely dissolved. The average microhardness value was 66.02 HV, significantly improving the work hardening problem of the corrugated tube. Simultaneously, excellent comprehensive fatigue performance and intergranular corrosion resistance were exhibited. The solid solution treatment process is more suitable for the manufacturing of corrugated pipes that require high formability and corrosion resistance, while the aging treatment requires strict temperature control to avoid the sensitization temperature zone. This study provides a theoretical basis for optimizing the heat treatment process of AISI 304 austenitic stainless steel corrugated pipes.

## 1. Introduction

Stainless steel bellows have the advantages of good flexibility, lightweight, corrosion resistance, fatigue resistance, and resistance to high and low temperatures. As a flexible pipe, it can absorb energy, reduce vibration, and compensate for displacement [1]. Therefore, stainless steel bellows are widely used in navigation, aerospace, instrumentation, nuclear, and petroleum industries [2,3,4,5]. Currently, the production and manufacturing of stainless steel bellows mainly involve molding, heat treatment, airtightness testing, and other multi-process completions. After molding, stainless steel bellows exhibit severe hardening and a decline in plasticity [6,7,8].

Due to the deformation process, the austenitic structure will have strain-induced martensite, twinning, stacking faults, and high-density dislocation generation. This results in high strength while reducing plasticity. The purpose of heat treatment is to improve plasticity while maintaining high strength. Strain-induced martensite formation is an effective method for optimizing the strength–plasticity balance [9,10].

Essoussi et al. [11] studied the effect of heat treatment on the mechanical properties of AISI 304 austenitic stainless steel, and the results showed that the strength and elongation of austenitic stainless steel increased after heat treatment, while the hardness decreased. Pham et al. [12] investigated the effect of parameters such as heating temperature, holding time, and cooling medium during heat treatment on the microstructure and hardness of austenitic stainless steel type AISI 304. Based on the hardness and organization characterization results obtained, the mechanism of strain-induced martensitic transformation in stainless steel after heat treatment was explained. Marikkannan et al. [13] improved the sensitization properties of stainless steel by a heat treatment process at 850 °C, 950 °C, and 1050 °C. AISI 304 stainless steel was heat-treated at 950 °C, followed by treatment at 650 °C, which proved to be beneficial for multi-pass welding of AISI 304 stainless steel joints. Samples annealed at 1050 °C for 2 h exhibited the highest hardness of 44 HRC. S.C.S.P. Krovvidi et al. [14] evaluated the tensile deformation behavior of AM350 stainless steel under two different heat treatment conditions: solution annealing and subcooling and tempering. Under solution annealing heat treatment conditions, the microstructure of AM350 stainless steel was predominantly austenitic. Under the supercooling and tempering heat treatment condition, the microstructure of AM350 stainless steel was mainly martensite. Compared to the solid solution annealed condition, the material in the subcooled tempered condition had higher yield and tensile strength and lower ductility at room temperature and 530 °C. Hence, many scholars have studied the microstructure and mechanical properties of stainless steel after heat treatment and found that different heat treatment conditions significantly affect the mechanical properties of stainless steel. This article explores the microstructure and fatigue properties of austenitic stainless steel corrugated pipes under different heat treatment conditions. Tumelo Moloi et al. [15] investigated the effect of high temperature on the mechanical properties and microstructure of DMLS Ti6Al4V (ELI) alloy. High-temperature annealing has a significant effect on the microstructure of Ti6Al4V (ELI) because the combination of α-flat noodles and β-flat noodles replaced the α-flat noodles. The effect of increasing the testing temperature was to reduce the average value of Vickers microhardness. The results indicated that temperatures between 175 °C and 325 °C had a significant impact on the mechanical properties and microstructure of the alloy.

This study focuses on AISI 304 austenitic stainless steel corrugated pipes (American Iron and Steel Institute, Washington, DC, USA) and systematically explores the influence mechanisms of aging treatment and solution treatment on their microstructure, mechanical properties, and corrosion resistance. The aims are to solve the performance contradictions faced by cold-formed corrugated pipes in practical applications, determine the optimal heat treatment process to improve the comprehensive performance of corrugated pipes, and provide theoretical support for optimizing their heat treatment processes.

## 2. Material and Sample Preparation

**Figure 1:**
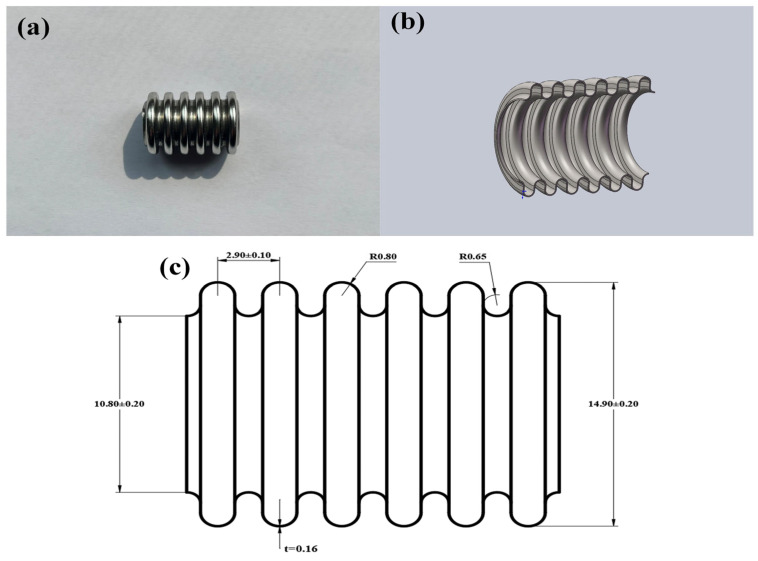
(**a**) Physical picture of corrugated pipe; (**b**) Sectional model of corrugated pipe; (**c**) Dimensional drawing of corrugated pipe.

The experimental research object was austenitic stainless steel corrugated pipes subjected to aging treatment and solution treatment conditions. The samples were taken from stainless steel corrugated pipes under two different heat treatment conditions. Figure 1a shows a physical image of the AISI 304 austenitic stainless steel corrugated pipe (American Iron and Steel Institute, Washington, DC, USA), Figure 1b shows a cross-sectional view of the corrugated pipe model, and Figure 1c shows the size diagram of the corrugated pipe.

The chemical composition of the stainless steel bellows was analyzed using an OLYMPUS handheld X-ray fluorescence spectrometer (Tokyo, Japan). The results are shown in Table 1.

The experimental samples of austenitic stainless steel corrugated pipes were obtained under the conditions of aging heat treatment and solution treatment. Sample #1 was subjected to aging heat treatment in a box furnace, which was placed at 600 °C and held for 120 min; sample #2 underwent solid solution treatment in the furnace. Solid solution treatment was carried out in a continuous heat treatment furnace at 1050 °C with a holding time of 240 s–300 s. The experimental setup consisted of three parts: heating, insulation, and cooling. The difference between the continuous heat treatment furnace and the box type treatment furnace was that the workpiece moved inside the furnace, and heating, insulation, and cooling were a continuous process. The cooling and insulation parts were integrated, with a dedicated pipeline in between to decompose ammonia gas. The cooling part was cooled with water at 0 to 5 °C. The cooling water did not directly contact the corrugated tube of the workpiece but instead rapidly cooled the corrugated tube through the cooling furnace tube and then slowly cooled to room temperature before being discharged from the furnace. The test samples are shown in Table 2.

## 3. Experimental Section

### 3.1. Microstructure Experiments

**Figure 2:**
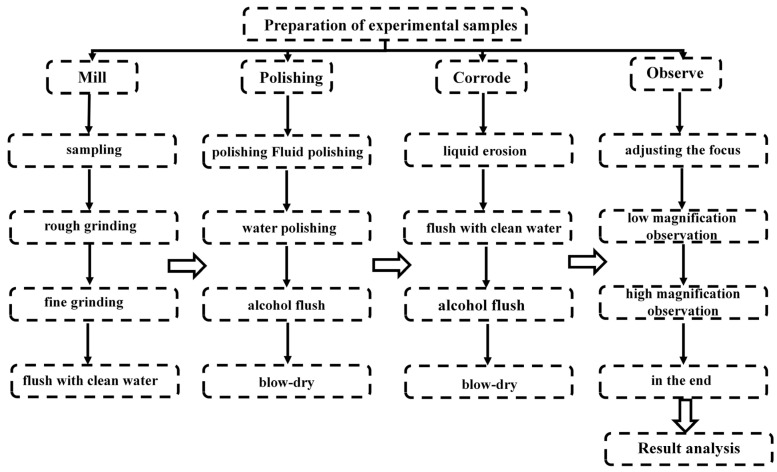
Preprocessing flowchart for microstructure experiments.

Microstructure experiments were conducted on corrugated tubes subjected to aging treatment and solution treatment at room temperature. The pre-treatment process of the corrugated pipes is shown in Figure 2.

The microstructure experiments included metallographic experiments, SEM, and EBSD. Figure 2 illustrates the preparation process for the metallographic experiments, where the sample preparation for SEM and EBSD testing was consistent with the preparation for the metallographic experiments. The external surfaces of samples #1 and #2 were pretreated, and SEM testing was conducted using a VEGA GM tungsten filament scanning electron microscope (Tescan, Kohoutovice, Czech Republic) at room temperature. The testing environment required a voltage of 20 kV and a beam current of 300 pa. The external surfaces of samples #1 and #2 were preprocessed, and EBSD testing was conducted using a MIRA LMS field emission scanning electron microscope (Tescan, Kohoutovice, Czech Republic) and QUANTAX electron backscatter diffractometer (QUANTAX, San Marcos, CA, USA) in a room-temperature working environment. The testing environment required an acceleration voltage of 20 kV and a beam current of 25 nA.

### 3.2. Fatigue Performance Experiments

**Figure 3:**
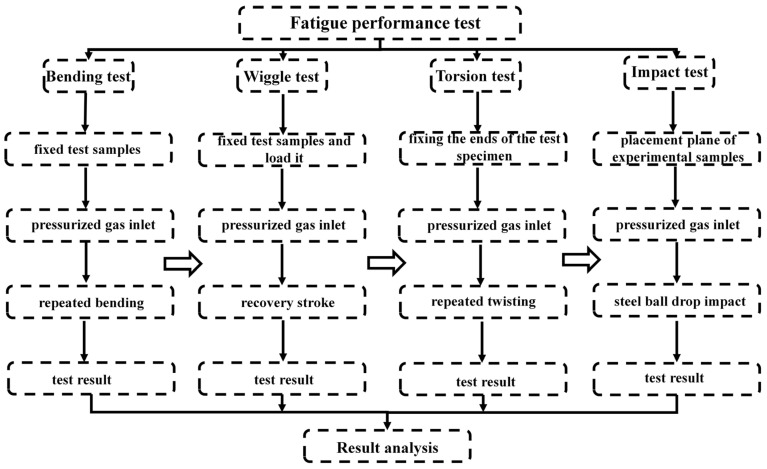
Flowchart of fatigue experiment program.

The fatigue life of stainless steel corrugated tube test samples under different heat treatment conditions in fatigue experiments is usually expressed in terms of the number of cycles. These data are key indicators for evaluating the fatigue performance of stainless steel corrugated pipes. Bending, twisting, swinging, and impact fatigue tests were conducted on austenitic stainless steel corrugated tube experimental samples numbered #1 and #2 at room temperature. Qualified standards were followed that complied with national standard GB/T41317-2022 [16]. The experimental procedure flow is shown in Figure 3.

**Figure 4:**
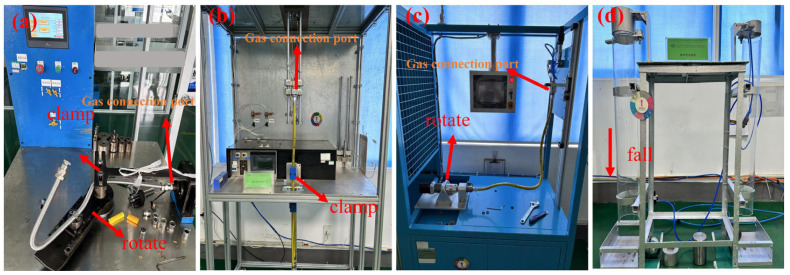
Bellows fatigue experiment instruments.

In this experimental process, fatigue experiments were conducted on stainless steel bellows under different heat treatment conditions to assess the fatigue performance and determine the fatigue life of the bellows. Bending Test: The stainless steel bellows experimental samples were fixed at one end in the bending fatigue test machine under an air pressure of 20 kPa. Positioned at a distance of no less than 100 mm from the fixed end, the samples were bent around a 30 mm diameter mandrel and subjected to repeated bending in both directions. This involved a second 180° bending, performed at a rate of 5 times/min, with uniform speed bending for 200 cycles. The cladding did not exhibit cracks, the bellows did not rupture, and the gas leakage did not exceed 10 mL/h, meeting the national standards for gas tightness. Qualified standards were followed that complied with national standard GB/T41317-2022 [16]. The bending testing machine is shown in Figure 4a.

Swing Test: The experimental prototype of the stainless steel corrugated pipe was fixed at one end of the swing test machine’s swing arm, with a 5 kg load suspended at the other end. Under an air pressure of 20 kPa, the swing arm swung from the middle position to +30°, returned to the middle position, swung again to −30°, and then returned to the middle position. The above comprised one cycle, and it swung for 10,000 cycles at the speed of 30 cycles per minute. There was no crack in the covering layer, no rupture of the bellows, and the gas leakage was not more than 10 mL/h, ensuring compliance with national gas tightness standards. The swing testing machine is shown in Figure 4b.

Torsion Test: One end of the stainless steel bellows experimental sample was fixed in the vertical position of the torsion test machine, while the other end was fixed in the horizontal rotating device of the same machine. Under an air pressure of 20 kPa, the rotating device moved from the middle position of the rotation to +90°, returned to the middle, and then to −90°. The above comprised one cycle of the rotation, which occurred 30 times per minute for 10,000 cycles. The cladding remained crack-free, the bellows did not rupture, and the gas leakage did not exceed 10 mL/h, meeting the national standard for gas tightness. The torsion testing machine is shown in Figure 4c.

Impact Resistance Test: At a pressure of 20 kPa, the stainless steel bellows experimental samples were placed flat on the hard plane at a distance of 1 m from the plane of the guiding device, and a steel ball was dropped in the center of the stainless steel bellows (2 kg). The cladding did not have cracks, the bellows did not rupture, and the gas leakage was not greater than 10 mL/h, in line with the requirements of gas tightness that meet the national standard qualified standards. In addition, during the experimental process, attention was paid to the use of the fatigue testing machine’s automatic shutdown device to avoid accidents. It was crucial to ensure the test piece was installed firmly to prevent lateral movement or tipping during the test. The impact resistance testing machine is shown in Figure 4d.

### 3.3. Hardness Test

Sample #1 of the corrugated tube was treated with aging and sample #2 of the corrugated tube was treated with solid solution. Firstly, pre-treatment was carried out on the observation surface, and microhardness testing was performed using the TMVS-1 digital micro Vickers hardness tester at room temperature. A load of 200 g was applied to the sample for 15 s. Five measurements from a random area were performed to reduce experimental error and avoid defects, and the average value was used with HV as the scale.

### 3.4. Corrosion Resistance Test

Corrugated pipes from the aging treatment and solution treatment were cut into 500 mm pieces as experimental samples. Both ends of the corrugated pipe were sealed and bent 30° in the middle. A solution of 20% sodium chloride, 1% sodium nitrite, and 79% distilled water was prepared and poured into a spherical open reactor. The corrugated tube sample was placed in the reactor with both ends facing upward, the power was turned on, the water pump was turned on to adjust the circulating water flow rate, and the time for turning on the electric heating jacket was set to 840 min. After cooling the solution, the corrugated tube sample was removed and 180° reverse bending was performed to test the airtightness and corrosion resistance of the stainless steel corrugated tube treated with aging and solid solution treatments.

## 4. Results and Analysis

### 4.1. Metallographic Results and Analysis

**Figure 5:**
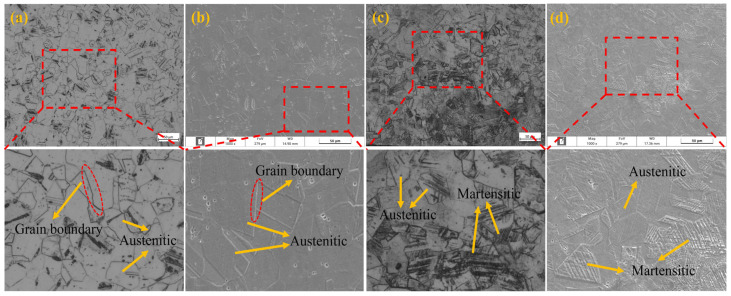
Microstructure of AISI 304 stainless steel and formed corrugated pipes.

Figure 5a shows the metallographic diagram of AISI 304 austenitic stainless steel, and Figure 5b shows the SEM image of AISI 304 austenitic stainless steel. Figure 5c shows the metallographic diagram of the corrugated tube after molding, and Figure 5d shows the SEM image of the corrugated tube after molding. From Figure 5a,b, it can be seen that the microstructure of AISI 304 stainless steel is mainly single-phase austenite, presenting a face-centered cubic (FCC) crystal structure. This structure contains a high content of elements such as nickel and chromium, which keeps austenite stable at room temperature. From Figure 5c,d, it can be seen that the crystal structure of the formed corrugated tube exhibits needle-like martensite. The process from Figure 5a,b to Figure 5c,d is the process of forming AISI 304 austenitic stainless steel into corrugated tubes through two spinning processes. During this process, austenitic stainless steel undergoes deformation, the average grain size of austenite decreases, the content of strain-induced martensite increases, and the dislocation density significantly increases. This results in a heterogeneous structure composed of high-density dislocations, deformation twins, strain-induced martensite, and austenite matrix. Under the combined action of strengthening mechanisms such as dislocation strengthening, fine grain strengthening, and especially martensitic strengthening, the strength and hardness of stainless steel corrugated pipes increase, while the plasticity decreases [17,18].

**Figure 6:**
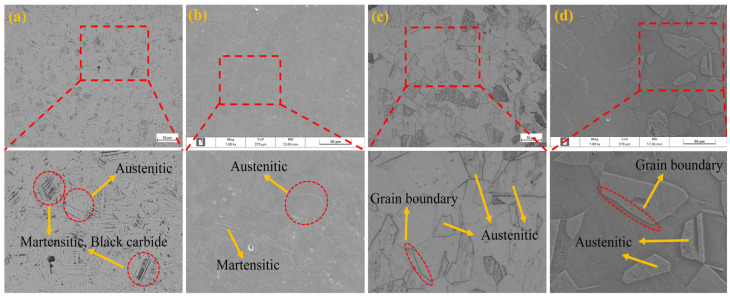
Microstructure diagrams of corrugated pipes treated with aging and solution treatments.

Figure 6a shows the metallographic diagram of the aged corrugated tube, and Figure 6b shows the SEM image of the aged corrugated tube. Figure 6c shows the metallographic diagram of the solution-treated corrugated tube, and Figure 6d shows the SEM image of the solution-treated corrugated tube. From Figure 6a,b, it can be seen that after aging treatment, the needle-shaped deformation-induced martensite still exists in the AISI 304 austenitic stainless steel corrugated tube and has not completely dissipated. At the same time, black carbon chromium compounds precipitate, forming a chromium-poor zone, significantly increasing intergranular corrosion sensitivity. From Figure 6c,d, it can be seen that after solid solution treatment, the microstructure of the AISI 304 austenitic stainless steel corrugated pipe is single-phase austenite without continuous carbides or intermetallic compounds. The deformation martensite generated by spinning disappears, and high-density dislocations also completely disappear.

The analysis of aged and solution-treated AISI 304 stainless steel corrugated pipes using electron backscatter diffraction (EBSD) technology focused on the changes in austenite and possible deformation-induced martensite, carbide precipitation behavior, and crystallographic characteristics.

**Figure 7:**
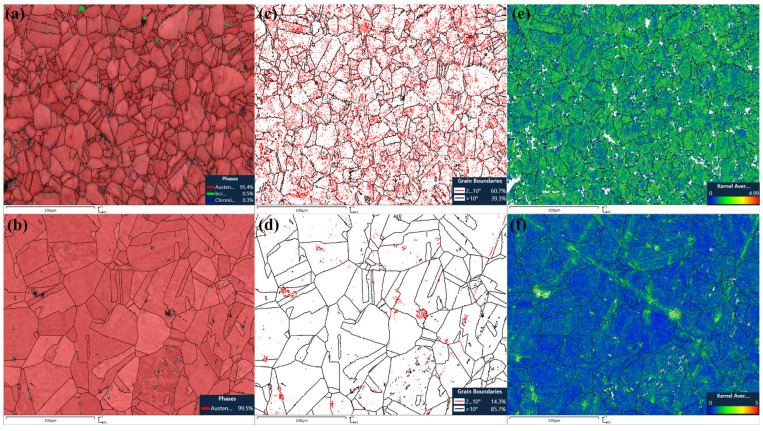
EBSD results of corrugated pipes treated with aging and solution treatments.

Figure 7a,b show the phase diagrams of age-treated sample #1 and solution-treated sample #2, respectively. From Figure 7a, it can be seen that the corrugated tube treated with aging is composed of 95.4% austenite, 0.5% martensite, and 0.3% precipitated carbon chromium compound. The martensite phase has not completely disappeared, and the formation of the precipitated phase can improve the strength of the material but reduce plasticity. At the same time, the appearance of the precipitated phase may damage the passivation film of the material, thereby reducing its corrosion resistance. The appearance of the precipitated phase will have a significant impact on the mechanical and corrosion properties of corrugated pipes. From Figure 7b, it can be seen that the corrugated pipe treated with solid solution is composed of 99.5% single-phase austenite structure. After solid solution treatment, the dissolution of the precipitated phase, such as carbides, and the homogenization of the austenite matrix reduce the diffusion channels and corrosion potential of the corrosive medium in the alloy, thereby reducing the corrosion rate. It can significantly improve the corrosion resistance of AISI 304 austenitic stainless steel corrugated pipes. After solid solution treatment, the corrosion resistance, plasticity, and toughness of the corrugated pipe is improved.

Figure 7c,d show the grain boundary diagrams of age-treated sample #1 and solution-treated sample #2, respectively. From Figure 7c, it can be seen that the content of small-angle grain boundaries in the age-treated corrugated tube is 60.7%, and the content of large-angle grain boundaries is 39.3%. The high proportion of small-angle grain boundaries reflects that the corrugated tube subjected to aging treatment has a metastable structure, which affects its corrosion resistance. It may also affect the mechanical properties of corrugated pipes, with increased strength but decreased toughness. From Figure 7d, it can be seen that the content of small-angle grain boundaries in the solution-treated corrugated tube is 14.3%, and the content of large-angle grain boundaries is 85.7%. After solid solution treatment, the proportion of large-angle grain boundaries in corrugated pipes may increase due to the dissolution of the precipitated phase, such as carbides, and the homogenization of the austenite matrix. This is because solution treatment promotes the migration and rearrangement of grain boundaries, allowing grains with larger orientation differences to form more large-angle grain boundaries. Large-angle grain boundaries have a significant impact on the mechanical properties and corrosion resistance of corrugated pipes. A higher proportion of large-angle grain boundaries means that the corrugated tube has better plasticity and toughness, as large-angle grain boundaries can more effectively hinder the slip of dislocations and the propagation of cracks.

The KAM value is a method for characterizing the supporting role angle of local faults in EBSD data analysis, which reflects the strain distribution and dislocation density in the local area of the material. A high KAM value usually indicates uneven strain distribution and high dislocation density in the region. Figure 7e,f show the KAM plots of age-treated sample #1 and solution-treated sample #2, respectively. From Figure 7e,f, it can be seen that the KAM value of the age-treated corrugated tube is higher than that of the solution-treated corrugated tube, indicating that the strain distribution of the age-treated corrugated tube is uneven and the dislocation density is higher than that of the solution-treated corrugated tube. The high KAM value reflects higher strength and hardness. A high KAM value may also lead to decreases in the plasticity and toughness of corrugated pipes after aging treatment. Uneven strain distribution and increased dislocation density may make corrugated pipes more prone to fracture under stress. Uneven strain distribution and increased dislocation density may become sensitive points for corrosion, thereby reducing the overall corrosion resistance of corrugated pipes.

### 4.2. Fatigue Performance Results and Analysis

**Figure 8:**
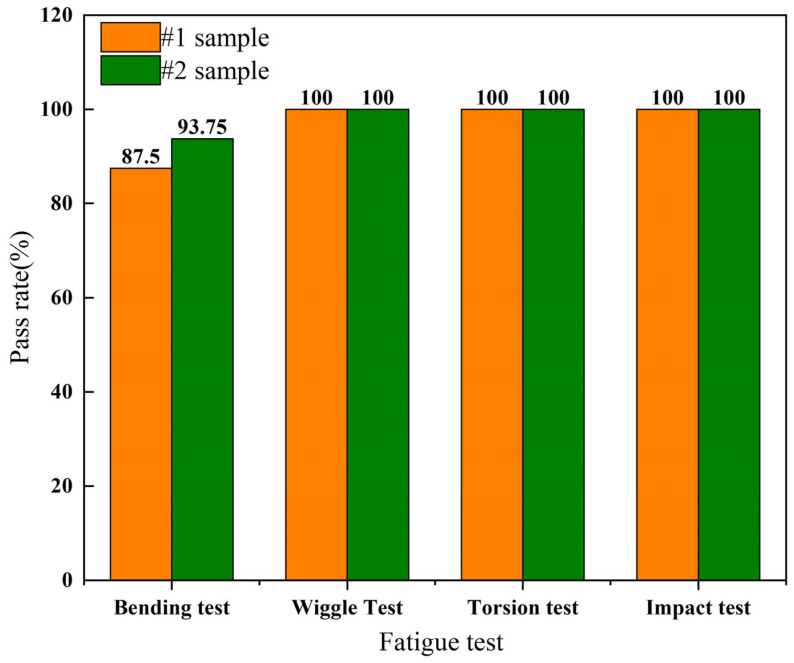
Qualification rates of fatigue tests for stainless steel corrugated tubes.

After aging treatment and solution treatment, austenitic stainless steel corrugated pipes were subjected to bending, swinging, twisting, and impact fatigue tests. The qualification assessment standards for austenitic stainless steel corrugated pipes were based on national standard GB/T41317-2022 [18]. The qualification rates of AISI 304 austenitic stainless steel corrugated pipes are shown in Figure 8.

From Figure 8, it can be seen that the qualification rates for bending, swinging, twisting, and impact of corrugated tube sample #1 after aging treatment are 87.5%, 100%, 100%, and 100%, respectively. The qualified rates for bending, swinging, twisting, and impact of solution-treated corrugated tube sample #2 are 93.75%, 100%, 100%, and 100%, respectively. Comparing the fatigue qualification rates of the two types of heat-treated corrugated pipes, the bending qualification rate of solution-treated corrugated pipes is higher than that of age-treated corrugated pipes.

### 4.3. Hardness Results and Analysis

**Figure 9:**
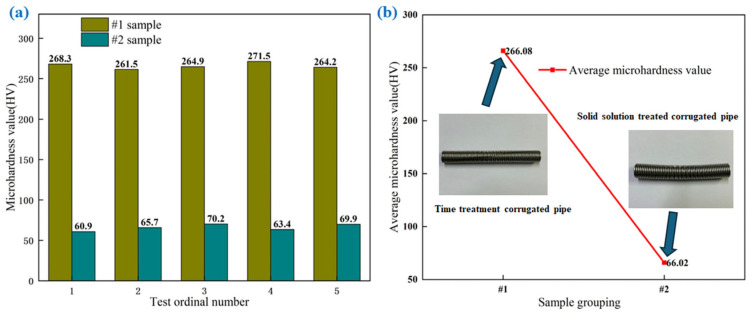
Microhardness values of corrugated pipes treated with aging and solution treatments.

Figure 9a shows the five microhardness measurements of the corrugated tubes treated with aging and solution treatments. Figure 9b shows the average microhardness values of age-treated corrugated pipes and solution-treated corrugated pipes. As shown in the figure, the average microhardness value of the stainless steel corrugated tube after aging treatment is 266.08 HV. After solid solution treatment, the average microhardness value of the stainless steel corrugated tube is 66.02 HV. During the aging process, small and dispersed carbides precipitate or hinder dislocation movement, but the hardness does not significantly decrease. Aging treatment does not improve the problem of work hardening in corrugated pipes. After solid solution treatment, due to the dissolution of carbides and the homogenization of the austenite matrix, the dislocations and residual stresses generated by cold working are eliminated, and the hardness of AISI 304 austenitic stainless steel corrugated pipes is significantly reduced, improving the work hardening problem of corrugated pipes.

### 4.4. Corrosion Results and Analysis

**Figure 10:**
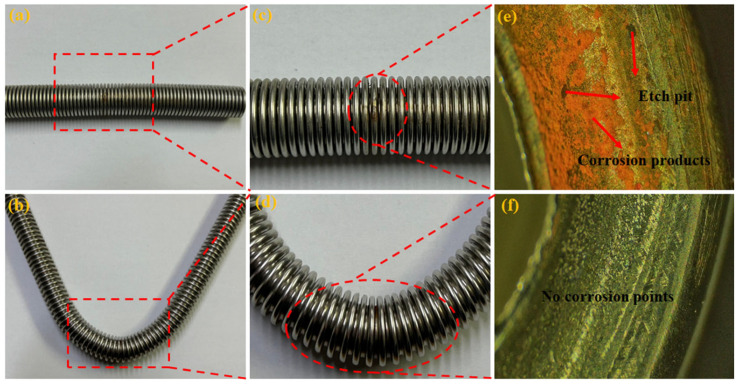
Corrosion results of corrugated pipes treated with aging and solution treatments.

By comparing the corrosion resistance of corrugated pipes under different treatment processes, a better stainless steel corrugated pipe treatment process was selected to improve the corrosion resistance of corrugated pipes. Figure 10a,b show the corrosion results of corrugated pipes subjected to aging treatment and solid solution treatment. Figure 10c,d show enlarged local views of the corrosion points, while Figure 10e,f show microscopic images of the corrosion points.

From the corrosion results, it can be seen that the corrugated pipe treated with aging has corrosion pits and red new organisms, resulting in corrosion behavior. This is because the age-treated corrugated tube is in the sensitization temperature range, and carbon atoms are easily combined with chromium elements to form carbides and precipitate along grain boundaries, forming a chromium-poor zone. The chromium content in the chromium-poor zone is lower than the critical value required for stainless steel corrosion resistance, thus becoming an anode in the corrosive medium and causing intergranular corrosion. For the corrugated pipe that undergoes solid solution treatment, there are no corrosion points or pits, and no corrosion behavior occurs. Solid solution treatment can eliminate or reduce carbides and other precipitates in alloys, making carbon atoms and alloying elements more evenly distributed in the matrix, optimizing the microstructure of materials and thus improving their stress corrosion resistance and corrosion resistance. Solution treatment is an effective means to improve the stress corrosion resistance of AISI 304 austenitic stainless steel corrugated pipes.

## 5. Conclusions

This study focuses on AISI 304 austenitic stainless steel corrugated pipes. It studies the changes in microstructure and properties of AISI 304 austenitic stainless steel corrugated pipes under aging treatment and solution treatment conditions. This study confirms that solution treatment is suitable for the heat treatment process of corrugated pipes. The conclusions are as follows:

1. Solid solution treatment effectively eliminated the deformation-induced martensite generated during the processing of corrugated pipes, restored a uniform austenite single-phase structure, and no obvious carbide precipitation was observed at the grain boundaries. After aging treatment, the deformation martensite generated by the spinning of the corrugated tube was not completely eliminated, and a small amount of carbides were precipitated in the microstructure.
2. After solution treatment, the austenite single-phase structure was restored, the grain boundary carbides were fully dissolved, and the grain size was homogenized. This process significantly reduced the hardness of the bellows and improved the work hardening problem associated with the forming process. After aging treatment, the second phase was precipitated, and the hardness of the bellows was not significantly reduced nor was the work hardening problem caused by the spinning of bellows improved.
3. The solid solution-treated corrugated tube exhibited excellent uniform corrosion and intergranular corrosion resistance in the corrosion resistance experiments, with no tendency toward corrosion. After aging treatment of the corrugated tube, the depletion of Cr elements at the grain boundaries significantly increased the sensitivity to intergranular corrosion, and the corrugated tube exhibited obvious corrosion behavior in the corrosion resistance experiments.
4. Solution treatment is more suitable for the heat treatment process of AISI 304 austenitic stainless steel corrugated pipes than aging treatment.

## Figures and Tables

**Table 1:** Chemical composition of stainless steel bellows (mass fraction) %.

	C	Si	Mn	P	S	Ni	Cr	N
Measured value	0.067	0.42	0.95	0.042	0.0052	8.45	18.46	0.045
GB/T 3280-2015	≤0.07	≤0.75	≤2.00	≤0.045	≤0.030	8.00~10.50	17.50~19.50	≤0.10

**Table 2:** Test samples.

Experimental Condition	Experimental Sample Set	Number of Samples
Aging treatment	#1	64
Solution treatment	#2	64

## Data Availability

The data cannot be made publicly available upon publication because no suitable repository exists for hosting data in this field of study. The data that support the findings of this study are available upon reasonable request from the authors.
